# An Accessory Muscle Originating From the Hyoid Bone and Inserting Into the Sternocleidomastoid Muscle

**DOI:** 10.7759/cureus.35708

**Published:** 2023-03-02

**Authors:** Philippe C Breda, Cristian Trache, Udo Schumacher, Thomas Graf von Rothenburg, Arne Böttcher

**Affiliations:** 1 Department of Otorhinolaryngology - Head and Neck Surgery, University Medical Center Hamburg-Eppendorf, Hamburg, DEU; 2 Department of Anatomy and Experimental Morphology, University Medical Center Hamburg-Eppendorf, Hamburg, DEU; 3 Department of Radiology, Radiologie Hoheluft, Hamburg, DEU

**Keywords:** surgical anatomy, neck dissection, digastric muscle, sternocleidomastoid muscle, accessory belly, irregular muscle

## Abstract

Irregular bellies and insertions into neck muscles have been described in the literature. To the best of our knowledge, a right accessory muscle originating from the hyoid bone and inserting into the sternocleidomastoid muscle has not been reported to date. Here, we report the case of a 72-year-old male patient with an irregular muscle originating from the lesser horn of the hyoid bone and inserting into sternocleidomastoid muscle fibers.

## Introduction

Surgical landmarks have to be borne in mind for any operation. Before the intervention, knowledge of anatomic variations is paramount for every surgeon as it might prevent severe complications. Irregular bellies and insertions into neck muscles have been described in the literature [1-5]. To the best of our knowledge, a right accessory muscle originating from the hyoid bone and inserting into the sternocleidomastoid muscle has not been reported to date. We describe our recent findings in a patient to alert surgeons to this rare variation in neck anatomy.

## Case presentation

**Figure 1:**
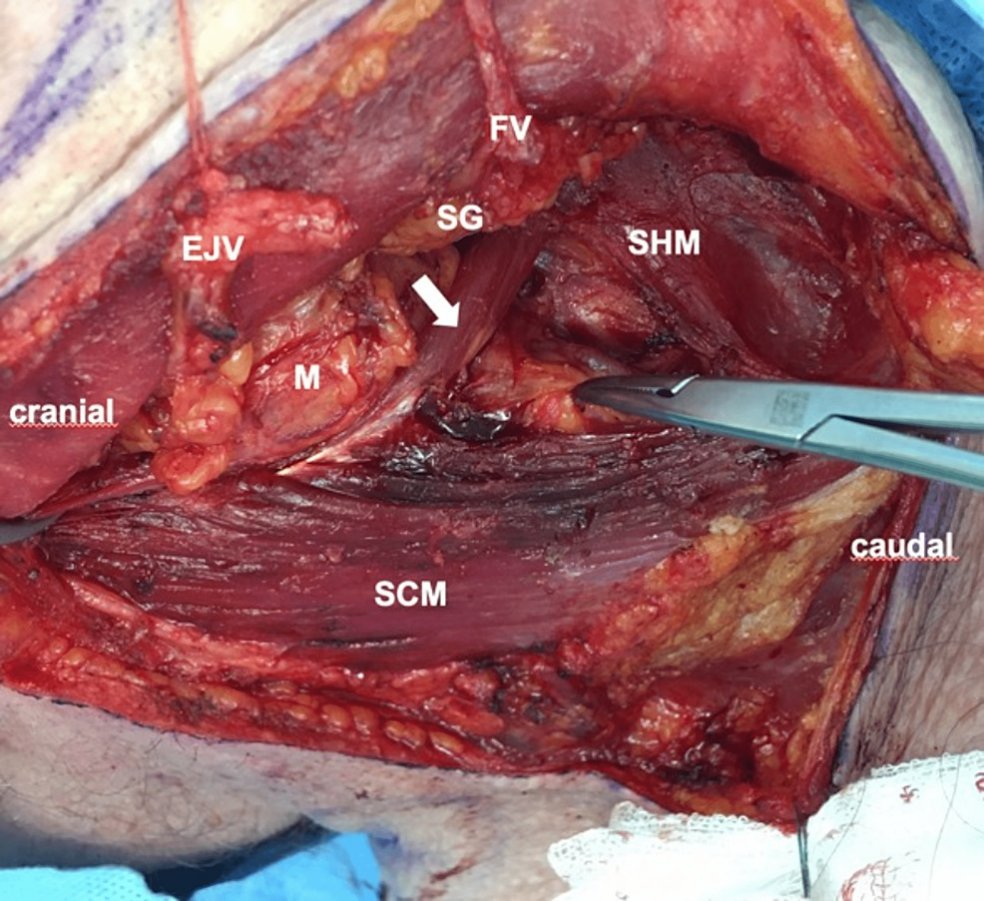
Intraoperative site in the right neck: presentation of the accessory muscle (arrow) extending from the hyoid bone to the upper sternocleidomastoid muscle. EJV, external jugular vein; FV, facial vein; M, metastasis; SCM, sternocleidomastoid muscle; SG, submandibular gland; SHM, sternohyoid muscle

A 72-year-old male patient diagnosed with oropharyngeal squamous cell carcinoma of the right palatine tonsil (cT1 cN1 cM0, p16+, HPV serotype 16+) underwent transoral tumor resection and modified radical neck dissection (levels I-V) on the right side, as approved after interdisciplinary tumor board consultation. Preoperative imaging included magnetic resonance imaging (MRI) of the neck and computed tomography (CT) of the neck, chest, and abdomen. After standard presurgical preparation, we commenced a modified radical neck dissection on the right side, with a hockey stick incision reaching from the mastoid tip to the cricoid cartilage followed by lifting the skin-platysma flap. The external jugular vein was dissected, ligated, and pulled outward. During dissection toward the submandibular gland using monopolar cautery, and ligation of the facial vein to identify the posterior belly of the digastric muscle, we detected an irregular muscle (width 1.2 cm; length 5.8 cm) originating from the lesser horn of the hyoid bone and inserting into sternocleidomastoid muscle fibers in an aponeurotic manner (Figure 1).

**Figure 2:**
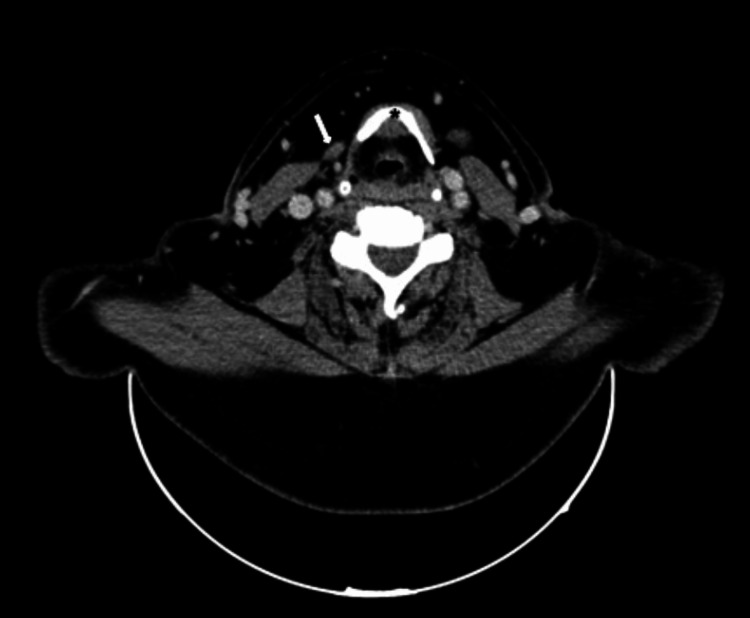
Contrast-enhanced computed tomography scan of the neck (axial view). Arrow, accessory muscle; asterisk, hyoid bone

**Figure 3:**
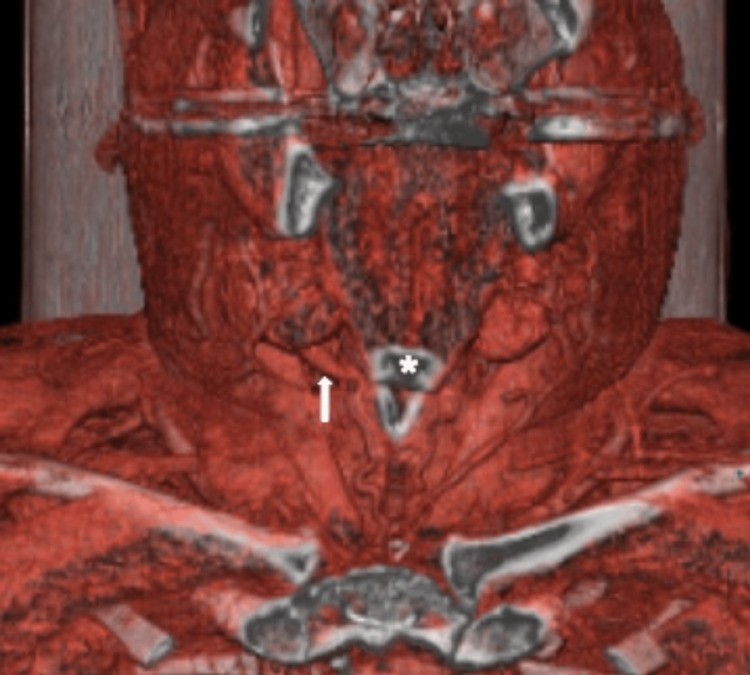
3-D computed tomography reconstruction (coronal view). 3-D, three-dimensional; arrow, accessory muscle in the right neck; asterisk, corpus of the hyoid bone

**Figure 4:**
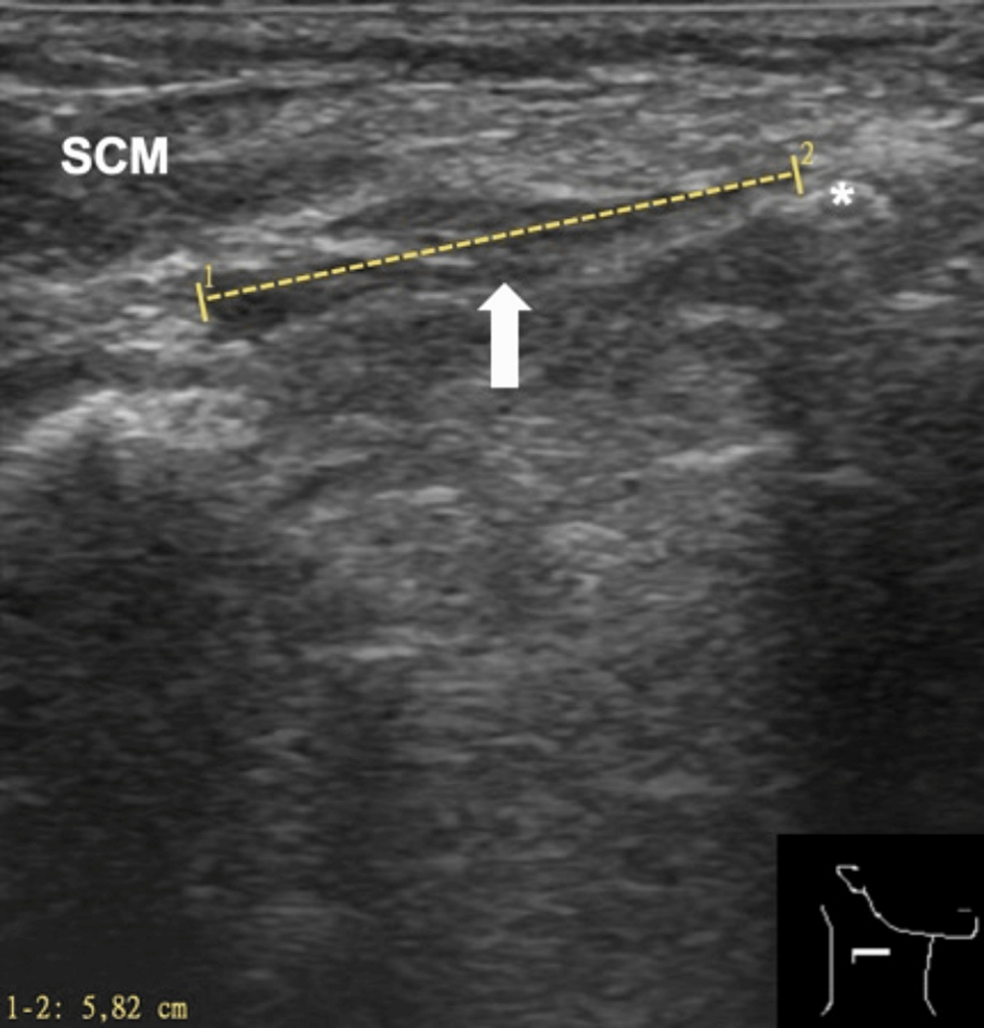
Ultrasound image of the right neck displaying the extent of the entire accessory muscle. Arrow, accessory muscle; SCM, sternocleidomastoid muscle; asterisk, corpus of the hyoid bone

One might have confused it with the posterior belly of the digastric muscle. After further exploration of the situs, we identified the regularly developed posterior belly of the digastric muscle underneath the accessory muscle. In addition to the intraoperative site (Figure 1), the location and route of the detected muscle could also be seen in a preoperative CT staging scan (Figure 2), a three-dimensional (3-D) CT reconstruction (Figure 3), and postoperative ultrasound scan of the patient's neck (Figure 4).

## Discussion

In the late nineteenth century, supernumerary bellies of the sternocleidomastoid muscle were described in postmortem anatomic examinations [6]. In this case report, we demonstrate a unique finding of an entire accessory neck muscle. During neck dissections, the surgeon was geared to the assumed location and route of important muscles such as the sternocleidomastoid and digastric muscles and the topography of neural and vascular structures. These structures, especially digastric and sternocleidomastoid muscles, facial veins, extra-lymphatic structures, mylohyoid muscle, and hypoglossal nerve, serve as landmarks during neck dissection [7]. Irregularities of these landmarks during neck surgery might be confused with a mass or lymph node in preoperative imaging. Therefore, surgeons should be aware of anatomic variations of neck muscles during imaging interpretation and differential diagnosis of neck masses. The occurrence of an accessory muscle, as described in this report, should be borne in mind during neck dissection to prevent damage to neurovascular structures or inadequate oncological management of levels IB, IIA, and IIB.

## Conclusions

Intraoperative identification of the digastric muscle is an important step during neck dissection. The irregular accessory muscle described here is an interesting variant, and head and neck surgeons should be aware of muscular variations when dissecting the neck. As it can mimic surgical landmarks, accessory muscles in levels IB, IIA, and IIB must not be mistaken for the posterior belly of the digastric muscle to prevent damage to important structures and maintain anatomical orientation during surgery.

## References

[1] Hsiao TH, Chang HP (2019). Anatomical variations in the digastric muscle. Kaohsiung J Med Sci.

[2] Sargon MF, Onderoğlu S, Sürücü HS, Bayramoğlu A, Demiryürek DD, Oztürk H (1999). Anatomic study of complex anomalies of the digastric muscle and review of the literature. Okajimas Folia Anat Jpn.

[3] Heo YR, Kim JW, Lee JH (2020). Variation of the sternocleidomastoid muscle: a case report of three heads and an accessory head. Surg Radiol Anat.

[4] Zhao W, Liu J, Xu J, Wang H (2015). Duplicated posterior belly of digastric muscle and absence of omohyoid muscle: a case report and review of literature. Surg Radiol Anat.

[5] Gruber W (1885). Supernumerärer Bauch des Musculus sternocleidomastoideus in der Richtung des hinteren Bauches des M. digastricus maxillae inferioris und abwärts von diesem zum Os hyoides. Archiv f Pathol Anat.

[6] Bonala N, Kishan TV, Sri Pavani B, Murthy PV (2015). Accessory belly of digastric muscle presenting as a submandibular space mass. Med J Armed Forces India.

[7] Lingeman RE, Shellhamer RH (1966). Surgical landmarks of the head and neck. Laryngoscope.

